# Microbial single-cell growth response at defined carbon limiting conditions†

**DOI:** 10.1039/c9ra02454a

**Published:** 2019-05-07

**Authors:** Dorina Lindemann, Christoph Westerwalbesloh, Dietrich Kohlheyer, Alexander Grünberger, Eric von Lieres

**Affiliations:** Institute of Bio- and Geosciences, IBG-1: Biotechnology, Forschungszentrum Jülich Jülich 52425 Germany e.von.lieres@fz-juelich.de +49-2461-61-3870 +49-2461-61-2168; RWTH Aachen University, Aachener Verfahrenstechnik (AVT.MSB) Aachen Germany; Multiscale Bioengineering, Bielefeld University Bielefeld Germany

## Abstract

Growth is one of the most fundamental characteristics of life, but detailed knowledge regarding growth at nutrient limiting conditions remains scarce. In recent years progress in microfluidic single-cell analysis and cultivation techniques has given insights into many fundamental growth characteristics such as growth homeostasis, aging and cell division of microbial cells. Using microfluidic single-cell cultivation technologies we examined how single-cell growth at defined carbon conditions, ranging from strongly limiting conditions (0.01 mmol L^−1^) to a carbon surplus (100 mmol L^−1^), influenced cell-to-cell variability. The experiments showed robust growth of populations at intermediate concentrations and cell-to-cell variability was higher at low and high carbon concentrations, among an isogenic population. Single-cell growth at extremely limiting conditions led not only to significant variability of division times, but also to an increased number of cells that did not pursue growth. Overall, the results demonstrate that cellular behaviour shows robust, Monod-like growth, with significant cell-to-cell heterogeneity at extreme limiting conditions, resembling natural habitats. Due to this significant influence of the environment on cellular physiology, more carefulness needs to be given future microfluidic single-cell experiments. Consequently, our results lay the foundation for the re-interpretation and design of workflows for future experiments aiming at an improved understanding of cell growth mechanisms.

## Abstract 2

Using microfluidic single-cell cultivation technologies and modelling we examined how single-cell growth at defined carbon conditions, ranging from strongly limiting conditions to a carbon surplus, influenced cell-to-cell variability.

## Graphical abstract

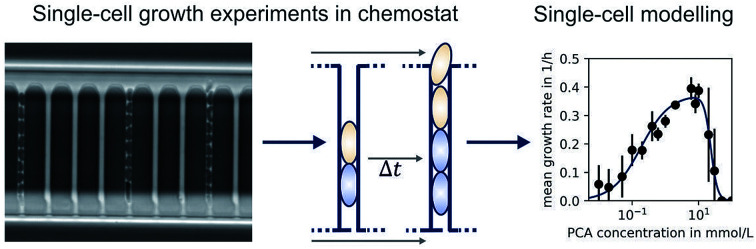

## 1 Introduction

Growth is one of the most fundamental characteristics of life. Especially the fast and dynamic proliferation of microbes is a popular and worthwhile subject for research and industry.

### 1.1 Quantification of microbial growth

In general, microorganisms adapt their growth to the environment in a way that benefits the entire population. Under stressful physio-chemical conditions, growth and division are reduced and energy is rather put into maintenance and other measures for survival.^1^ More habitable conditions induce accelerated growth for developing larger populations which are potentially more robust towards unfavorable environments.

Under constant environmental conditions the growth rate of a population will converge towards a value solely determined by the current environmental parameters, independent of the population's history. Generally only one of these parameters, for example nutrient concentration, temperature, pH or osmotic pressure, is limiting. The relationship between growth rate and the particular limiting parameter, acting as the growth-controlling factor, can be measured by systematically varying the limiting parameter. The result is a growth kinetic which stretches from no growth, usually close to or at the absence of the limiting component, across a range of linear dependence of growth on the nutrient concentration up to saturation and ends with inhibition or toxicity. The situation of a single substrate being growth limiting can be studied by using a single carbon source that is exclusively metabolized and thereby determines growth. Monod has provided a mathematical description of a growth kinetic, as long as only one parameter is limiting and no inhibitors are present.^2^ Depending on this carbon source, a bacterial culture will show a substrate-specific growth kinetic with a certain maximum growth rate (*μ*_max_) and substrate concentration (*K*_s_) at which 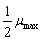 is reached. For a quantitative and meaningful analysis of *μ* several preconditions have to be fulfilled by the respective cultivation device and measurement technique. Firstly, the provision of a stable and simultaneously highly versatile and controllable environment over a wide range of nutrient concentrations, pH values or temperatures. Secondly, the maintenance of steady state growth over a longer period of time and thirdly, a coupled online measurement that records data in high-resolution, real-time and non-invasively.

### 1.2 Macrofluidic cultivation systems

**Fig. 1:**
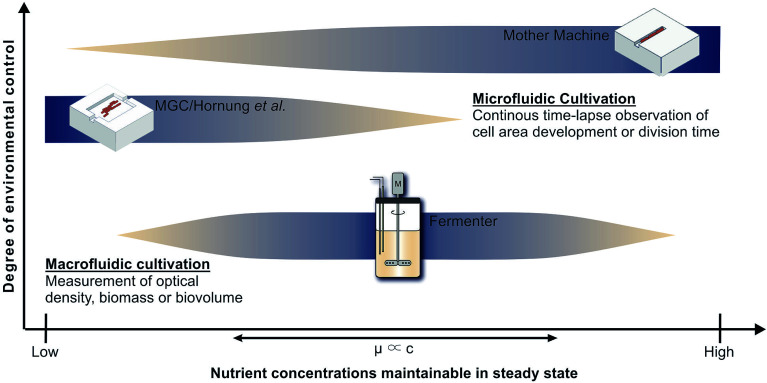
Applicability of cultivation devices for nutrient screenings and growth analysis in chemostat. Top: Mother machine (MM);^22^ middle: monolayer growth chamber (MGC) used in combination with the simulations of Hornung *et al.*;^16^ bottom: table-top fermenter.

Cultivation devices applied to establish growth kinetics can be divided into macro- and microfluidic operating systems (see Fig. 1). Among the most commonly used devices are table-top fermenters, baffled or non-baffled cultivation flasks and miniature bioreactor systems (MBRs) in micro-well plate formats.^3,4^ Apart from their sizes, the most striking differences between these macrofluidic devices are the throughput capacities, costs of maintenance and operating principles. Cultivation flasks and MBRs are mainly operated in batch mode. The downside of this operating mode is that the maximum growth rate occurs just for a short time until nutrients become limiting or inhibiting by-products accumulate. Only with additional equipment and modifications a chemostat or turbidostat cultivation, required for steady state growth, can be achieved.^5,6^ As opposed to this, table-top fermenters are usually equipped to operate as chemostat. They provide online measurements of optical densities and sample sizes suitable for chemical and gas analyses, which may be used, *e.g.*, for ^13^C flux analysis. However, a constant removal of culture supernatant and replenishment with fresh medium at this scale bears the risks of contamination and introduction of heterogeneities,^7^ while it involves high expenses and an elaborate experimental setup. Fig. 1 shows how very low growth rates, expected for low and high nutrient concentrations, are difficult to measure in such devices. When cells stop growing they will be flushed out, preventing the measurement of inhibitingly high nutrient concentrations. For low nutrient concentrations the cell density should also be low to keep the reactor gradient free. However, online measurement of such cell densities is still very difficult.

### 1.3 Microfluidic cultivation systems

As alternative to the classical macrofluidic cultivation, microfluidic devices come in a wide variety of designs. They feature several technical properties:

(1) Precisely controlled chemostat cultivation that minimizes the occurrence of concentration and temperature gradients due to fast transfer of mass and heat.

(2) Applicability of a wide range of media, pH, temperatures, *etc.*

(3) Automated, noninvasive and constant observation in high spatio-temporal resolution with time-lapse microscopy, which is robust against optical properties of medium components.

(4) A typically very high structure density with regard to cultivation sites, which can be utilized for high-throughput by/through parallelization of experiments with high cell densities.

(5) Laminar flow without turbulences, quantified by a low Reynolds number.

(6) Mixing of solutes dominated by diffusion.

Microfluidic cultivation devices can be classified by the degree of freedom in which cells proliferate.^8^ In regard of environmental maintenance, experiment duration and the acquisition of growth rates, they exhibit certain differences.

The highest degree of isolation for single-cell analysis is achieved by negative dielectrophoresis.^9^ This technically rather demanding setup for non-contact cell traps is driven by an electric field in which a few cells can be held over several generations with exceptional environmental control, restricting the dimensions available for translational movement practically to zero. However, as side-effect the trap creates heat, potentially influencing cell physiology.^10–12^ It is also difficult and time consuming to collect data for large numbers of cells, since the number of trapped and thereby observable cells is comparably limited.

Monolayer growth chambers restrict cell growth to a single layer and enable monitoring of classical colony formation and expansion in two dimensions^13,14^ with growth rates often defined by area increase. This limits the experiment duration until chambers, often sized approx. 50 × 50 μm, are overgrown. Chambers with very wide entrances can be used to create stable conditions for long times, but nutrient gradients will form at low concentrations.^15^ Therefore Hornung *et al.* have used a model to describe the relationship between nutrient uptake, cell growth and movement and have been able to determine growth kinetics and uptake rates for *C. glutamicum* at limiting C-source concentrations.^16^ However, high nutrient concentrations lead to high growth rates and fast cell movement, which also creates significant challenges for the image analysis that is required to determine the desired growth rates. Therefore Fig. 1 indicates the combination of monolayer growth chambers and modeling to be best for low nutrient concentrations.

**Fig. 2:**
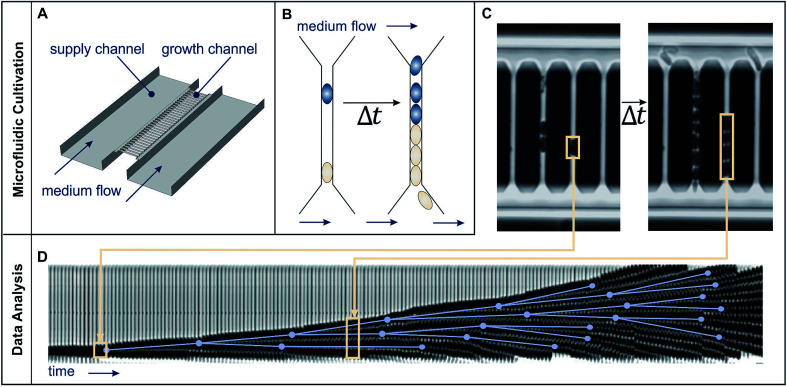
Microfluidic single cell cultivation device and image analysis. (A) Schematic draft of medium flow, supply and growth channels. (B) Schematic cell proliferation in a growth channel over time. (C) Phase contrast images of growth channels shortly after cell seeding and after 2.6 h cultivation. (D) Image analysis: growth channels are automatically cut out (beige frames) and later sequentially arranged to form a kymograph. Manually detected cell division events (blue dots), connected by the lines representing cell life time, created using the *ground truth mode* of molyso.^20^

The present study deploys the microfluidic mother-machine (MM) design, which restricts cell division in a linear, one-dimensional manner. Excess cells are removed with the medium flow at the open ends of each growth channel (see Fig. 2). Thereby, experiment duration becomes theoretically infinite, enabling high resolution measurement of *μ*_max_ while excluding effects of the preculture. In regard of data acquisition and analysis, cell tracking and lineage recreation are facilitated and more detailed compared to monolayer growth chambers.^17–20^ Until now the MM design has been used for studies concerning limited bacterial growth, physiology, dynamic gene regulation or cell-size control and homeostasis.^17–19,21^ With a growth channel length of 15 to 20 μm and a diameter of 1 μm, nutrient gradients only appear at very low concentrations. Under such conditions it becomes difficult to derive, calibrate and validate appropriate models, as it is possible for monolayer growth chambers.^16^ Investigations at very high nutrient concentrations on the other hand are very well possible, since the cells are caught within the growth channels and can not be flushed out even if they do not grow (see Fig. 1).

The choice of cultivation device potentially influences the measurement, for example *via* spatial restriction of cellular movement. However, Dusny *et al.*^12^ have compared different microfluidic cultivation devices with regard to the growth of *C. glutamicum* and while they have reported differences regarding the snapping motion connected to cell division they have found very similar growth rates for substantially different degrees of confinement. Therefore we expect our results to be valid across different cultivation devices and scales.

### 1.4 Cell-to-cell heterogeneity

Until now macrofluidic devices and bulk measurement techniques have been used more frequently to determine microbial growth *via* biomass, biovolume or optical density (OD).^23^ Meanwhile, growth rates acquired in the microfluidic single-cell cultivation can be based on doubling times measured with the help of time-lapse microscopy.^13^ The additional information gained on a single-cell level allows to determine not only *μ*_max_ very precisely but also gives information on cell-to-cell heterogeneity.

Heterogeneity in bacterial populations has so far consistently been observed in biofilms, pathogenic communities, natural environments and bio-processes.^24–26^ Especially under stressful conditions, sub-populations with different metabolic and phenotypic characteristics and abilities appear to be beneficial for the survival of a population.^27–29^ The resulting “noise” in bacterial populations has proven to be a major source of heterogeneity even in isogenic populations grown in the same environment.^25,30^ The particularly detailed representation of proliferation in microfluidic single-cell cultivation adds a great quality to growth kinetic analysis by including cell-to-cell heterogeneity.

### 1.5 *C. glutamicum* and PCA

We cultivated *C. glutamicum* with protocatechuic acid (PCA) as limiting carbon source in defined CGXII mineral medium to study growth and cell-to-cell heterogeneity. *C. glutamicum* is the work-horse of modern biotechnology especially in large-scale production of amino acids, vitamins and polymers.^31–34^ PCA has traditionally been added to defined media for *C. glutamicum*, for example CGXII,^35^ as iron chelating compound to reduce lag phases and accelerate growth in batch cultivation.^36^ However, studies by Unthan *et al.* revealed that PCA has additional effects on growth in CGXII.^37^ A microfluidic cultivation approach showed growth of *C. glutamicum* on PCA without the addition of glucose or other carbon sources. Unthan *et al.* also performed batch cultivation growth studies with *C. glutamicum* on CGXII medium with increased PCA concentrations and observed biphasic growth behaviour. Further GC-ToF-MS, LC-MS/MS and transcriptome analysis revealed a parallel utilization of PCA as carbon and energy source,^37^ in addition to its iron chelating purpose. Both the microfluidic experiments by Unthan *et al.* as well as macrofluidic turbidostat cultivation at low cell densities with a similar mineral medium by Bäumchen *et al.* resulted in growth rates higher than batch cultivation with CGXII.^6,37^

Merkens *et al.* had already reported that PCA can be used as sole carbon source for *C. glutamicum*.^38^ Merkens *et al.* reached growth rates of 0.14 h^−1^ in microtiter plates and shaking flasks using CGXII as growth medium.^38^ They also discussed the importance of co-metabolization of both carbon sources in minimal media for *C. glutamicum*, usually PCA and glucose, and came to the conclusion that co-metabolization is likely.^38^ Subsequent experiments with CGXII but without glucose used varying levels of PCA as limiting carbon source in microfluidics.^15,16^

### 1.6 Scope of this study

Even though the microfluidic single-cell cultivation in growth channels enables high resolution measurement of bacterial growth, to the best of our knowledge, nutrient dependent kinetics, especially the affinity constant *K*, have so far not been determined using this design. In the course of this study *C. glutamicum* was cultivated on a modified version of the defined CGXII mineral medium with protocatechuic acid (PCA) as sole carbon source. Growth rates were determined in a constant perfusion cultivation mode of over 40 h based on single-cell doubling time distributions for PCA concentrations from 0 mmol L^−1^ to 100 mmol L^−1^. Growing *C. glutamicum* in steady state for several hours allows to exclude effects of pre-cultivation.

We expanded the use of an existing technology platform towards a new application, the easy and precise experimental acquisition of growth kinetics. The methods applied here serve as model for the investigation of the influence of media components on cellular physiology. The microfluidic single-cell cultivation system was taken to its operating limits and the range of available growth data for PCA as substrate was expanded significantly compared to earlier data from Hornung *et al.*^16^ For the first time the full concentration range including single-cell division age distributions was covered.

## 2 Material and methods

### 2.1 Microfluidic single-cell cultivation setup

The present study employs the mother-machine (MM) design using a growth channel width and height of 1 μm in dead- and open-end versions modified from Wang *et al.*^22^ and Schendzielorz *et al.*^39^ (see Fig. 2 and 8, ESI†). It provides quasi-one-dimensional growth channels to restrict cell proliferation along one axis (see Fig. 2C and 8(c, d), ESI†). The openings face towards supply channels where fresh medium flows with a high velocity (several mm s^−1^), ensuring a constant supply of fresh medium to the growth channels and washing out of metabolic products (see Fig. 2A and B). On each chip there are four separate inlets, each of them connected to an array of 10 times 31 mother machines with 30 growth channels each. Fig. 2 summarizes the structure of the microfluidic single cell cultivation device and the methods for image analysis.

### 2.2 PDMS chip production

The wafer production took place in a clean room facility using soft-photolithography.^40^ For a detailed description of the manufacturing process the reader is referred to Grünberger *et al.*^41^ The wafers were covered with a degassed 1 + 10 mixture of cross linker and polymer base (Silicone Elastomer Kit #184, Dow Corning, Midland). After curing at 80 °C for at least 1.5 h, non-polymerized elements were removed by a chemical treatment with one *n*-pentane and two acetone steps for 1.5 h each. Individual perfusion channels of each chip were made accessible by punching inlets and outlets through the PDMS (punching tool diameter 0.75 mm, WPI, Sarasota). Finally, clean and dry chips were bonded to glass substrates (thickness 170 μm, Schott, Mainz) by surface activation in an oxygen plasma generator (Diener, Ebhausen) for 25 s at 0.8 mbar.

### 2.3 Strain & precultivation

The *Corynebacterium glutamicum* wild type (ATTC13032) was used from a single Roti®-Store-Cryovial (Carl Roth, Karlsruhe) throughout this study. Precultivation was performed in two steps, both using standard CGXII minimal medium, introduced by Keilhauer *et al.*,^35^ with 40 gL glucose according to Unthan *et al.*^37^ The CGXII medium contained per liter of distilled water: 20 g (NH_4_)_2_SO_4_, 5 g urea, 1 g K_2_HPO_4_, 1 g KH_2_PO_4_, 0.25 g MgSO_4_·7H_2_O, 42 g 3-morpholinopropanesulfonic acid (MOPS), 13.24 mg CaCl_2_·2H_2_O, 10 mg FeSO_4_·7H_2_O, 10 mg MnSO_4_·H_2_O, 1 mg ZnSO_4_·7H_2_O, 0.313 mg CuSO_4_·5H_2_O, 0.02 mg NiCl_2_·6H_2_O, 0.2 mg biotin, 30 mg PCA adjusted to pH 7. After 24 h cultivation, the second preculture was inoculated to an optical density (OD) between 0.2 and 0.3. Incubation was performed at 30 °C and 120 rpm (incubator, GFL, Burgwedel) in baffled flasks with 20 mL medium. As soon as an OD of approx. 1.5 was reached the second preculture was seeded into the microfluidic cultivation device.

### 2.4 Microfluidic cultivation

Before cell seeding the chip was flushed with the respective medium for several hours to facilitate the seeding process.^42^ A constant medium supply was ensured by a low pressure syringe pump (Cetoni, Korbussen) and tubing (Tygon AAD04103, Saint-Gobain Performance Plastics, Akron, OH, USA) with proper junctions (Precision tips, Nordson, Erkrath) to the chip and medium supply syringes (ILS, Fürstenfeldbruck) respectively. The on-chip-cultivation was performed at 30 °C with a medium flow of 500 nL min^−1^ and a modified version of the standard CGXII minimal medium. In this medium glucose was omitted and PCA as a sole carbon source was used in concentrations between 0 and 100 mmol L^−1^. Medium was sterile filtered before usage to reduce particle contamination (pore size 0.2 μm, Sarstedt, Nümbrecht). Cells grew in steady-state under chemostat conditions for approx. 40 h, monitored *via* time-lapse microscopy in a resolution of 5 min. During every experiment one of the four channels of the chip was used to cultivate a reference using standard CGXII medium containing PCA and glucose.

### 2.5 Data analysis

Microscopy was performed with a Nikon Ti-E Microscope and a high resolution camera from Zyla (sCMOS, Andor, Belfast). Automated time-lapse microscopy in phase contrast used a CFI Plan Apochromat λ100× oil objective (NA 1.45; W.D.0.13; Nikon, Tokyo). Positions of growth channels with a high seeding efficiency were selected manually. For data extraction, the mother-machine analysis tool (molyso) by Sachs *et al.* was used in the *ground truth mode* (see also Fig. 2).^20^ The program created a kymograph, an image of one individual channel at all recorded time points aligned in sequential order. Due to varying image qualities the automated mode of molyso was prone to false detection of division events. To guarantee accurate data, proliferation was marked manually by determining first and last appearance of each cell, and the division age was subsequently derived from the resulting data.

**Fig. 3:**
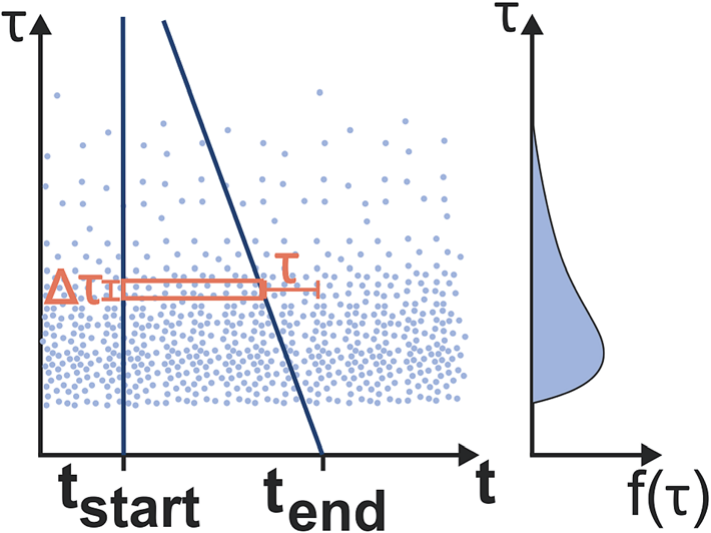
Determination of the division time distribution *f*(*τ*) from the limited experimental time window: The left graph shows the single cell division events with the cell division time *τ* plotted at the beginning of the cell life over time *t*. The observed time window reaches from *t*_start_ until *t*_end_. The right graph shows the underlying distribution of cell generation times. The events (light blue dots) between the dark blue lines can be observed. Therefore the width of the observed time window (red box) with the height Δ*τ*, which is determined by the image frequency of the microscope, depends on the cell division time *τ*.

To exclude potential effects from the preculture, all data points of cell division events starting before 15 h after experiment start, *t*_start_, were discarded. Experiments with less than 5 division events were marked as growth rate zero. Experiments with more than 5 and less than 50 events were ignored as the number of data points was considered too low to be representative of a population growth rate. The non-zero growth rates were fitted using eqn (1) for deriving colony growth rates from single-cell division time distributions:^13,43^1
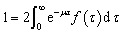
here *f*(*τ*) describes the probability density function of the single-cell generation times, *μ* the colony growth rate and *τ* the single-cell generation time. To approximate the integral over the probability density function from the available data the division events within a time span were counted and divided by the sum of all division events. Fig. 3 shows how the number of division events which can be observed during an experiment is directly influenced by the generation time. The left plot shows observed events, where the single cell generation time is plotted over the starting time of the single-cell life. The right plot shows the corresponding probability density function or distribution of single-cell generation times, which is assumed to be stable over time. For any experiment with limited duration from *t*_start_ until *t*_end_, the observable cell division events fall between the two blue lines on the left plot. The events outside of the right blue line cannot be observed because if the cell life starts too late, its end, marked by cell division, will not be observed within the experiments' duration. This means there is a higher probability to observe shorter cell generation times. The observation time length for a given cell generation time *τ* is shown as a red square, which has the length *t*_end_ − *t*_start_ − *τ*. To remove this bias from the data we introduced a correction factor *c*_*τ*_:2
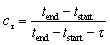


The correction factor scales the observed cell generation times so that if a long generation time *τ*_1_ and a short generation time *τ*_2_ have the same probability, we can also expect the same number of observed events for both generation times. Eqn (1) can be converted into a sum to approximate the probability distribution:3
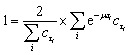


### 2.6 Growth rate modeling

After acquiring the population growth rates, we used two different models to describe them depending on the PCA concentration in the medium. A single step kinetic is described by the common Monod model multiplied with a Hill-type inhibition term with exponent *n*_I_ (see eqn (4)):4
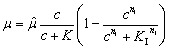
with the growth rate *μ* in h^−1^, the maximum growth rate *μ̂* in h^−1^, the PCA concentration *c* in mmol L^−1^, the half-velocity constant *K* in mmol L^−1^, and the constant for the inhibition term *K*_I_ in mmol L^−1^.

As the existing literature indicates several uptake systems working in parallel, we expanded the growth model by adding a second Michaelis–Menten term (see eqn (5)), which is equivalent to two kinetics working in parallel.^44^5
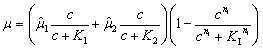
here *μ̂*_1_ and *μ̂*_2_ are the maximal growth rates of the two kinetics, and *K*_1_ and *K*_2_ the respective half-velocity constants. The data were processed using python version 3.6.7 with the pandas package version 0.23.4, the population growth rate equations were solved using fsolve and the model curves were fitted using the curve_fit function from the scipy.optimize package version 1.1.0. For the fit we used all individual results of the experiments and not the mean value for each concentration. For illustration, the mean and variances of the distributions were calculated using stats.weightstats from the statsmodels package version 0.9.0.^45^

## 3 Results and discussion

### 3.1 Validation of the operating range

Microfluidic single-cell cultivation enabled us to determine steady-state growth on a single carbon source over a wide concentration range of nearly 4 orders of magnitude. High amounts of fresh growth medium compared to microbial biomass ensured stable and well defined environmental conditions in most of the applied nutrient concentrations over the whole course of the experiments (see Fig. 12–17, ESI† for growth rates *vs.* growth channel position along the supply channel). The standard cultivations (see also Fig. 9, ESI†) showed good reproducibility of the microfluidic cultivation, as growth rates and single-cell division time distributions were in good agreement.

**Fig. 4:**
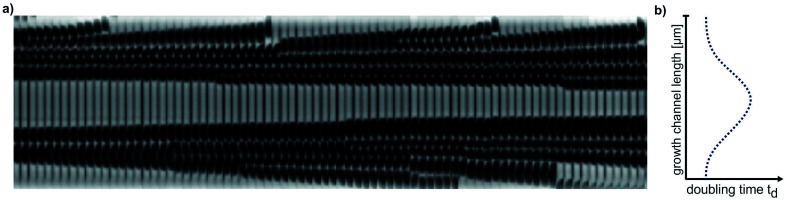
Spatial heterogeneity along a growth channel. (a) Kymograph of cell proliferation in 0.01 mmol L^−1^ PCA. (b) Schematic graph of doubling time distribution along the growth channel.

However, at concentrations of 0.01 mmol L^−1^ to 0.02 mmol L^−1^, spatial heterogeneity occurred along the growth channel (see also Fig. 4). Cells close to the channel opening proliferated, while cells located more centrally grew significantly slower or even stopped dividing. Based on the work of Hornung *et al.*, the boundaries of the operating range of our system were assessed.^16^ Hornung *et al.* used a specific chamber geometry and a mass balance based approach to fit kinetic parameters of *C. glutamicum* to experimental data. They reported an uptake rate per single cell *u*_∞_*ḡ*:6
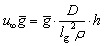
7
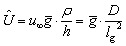
with *D* = 2.8 × 10^−10^ m^2^ s^−1^ for PCA in water,^46^ the fitted parameter *ḡ* = 19.9 μmol L^−1^ and *l*_g_ = 3.78 μm for a Monod-kinetic. An estimate of *ρ* = 0.66 μm^−2^ for the cell density led to good model predictions. With a given chamber height of *h* = 0.8 μm this results in a single cell uptake rate of *u*_∞_*ḡ* = 4.72 × 10^−19^ mol s^−1^. We can further estimate a colony volume related uptake of *Û*= 0.39 mol s^−1^ m^−3^.

This volume related uptake can be used to find the medium inlet concentration 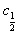 at which the cells in the center of the growth channel, open at both ends, would have only half of the medium inlet concentration (see also ESI†):8
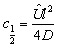


As our growth channels have a length *l* of *ca.* 20 μm, we calculate a critical PCA concentration of 
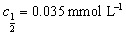
. The predicted diffusive transport limitation presumably explains the observed spatial heterogeneity along the growth channel for concentrations below 0.05 mmol L^−1^. For some of the experiments at low concentration no division events at all were observed, further underscoring the heterogeneity of the phenotypic reaction to such challenging conditions.

At higher concentrations (20 and 30 mmol L^−1^), potential toxicity leads to a higher metabolic burden and thus to slower growth and death of the cells under investigation. Since the resulting long generation times were not fully covered by the experiment duration, the length of detectable doubling times was obviously limited and results may be shifted towards a lower mean value, marking the upper boundary of our technology. Similar to low concentrations the experimental results also varied strongly from experiment to experiment, some experiments without any growth at all, while few cells grew comparatively fast.

We also investigated the percentage of all channels with captured cells, that contained cells dividing within the first 15 h of the experiment (see Fig. 10, ESI†). Interestingly for all concentrations below 50 mmol L^−1^, between 80% and 100% of all channels showed cell divisions, while concentrations of 50 mmol L^−1^ and above led to inactivity. The ratio of channels containing dividing cells appeared to be independent of growth channel type and concentration (see also Fig. 11, ESI†). A possible interpretation is that for low concentrations cells are able to use stored nutrients from the preculture, but for high concentrations the bacteriostatic effect of PCA is too strong and effects all cells equally.

### 3.2 Kinetic

In our experiments *C. glutamicum* was not able to initiate growth in microfluidic single-cell cultivation without PCA, even in the presence of glucose. For the same CGXII standard medium with glucose but without PCA in a 20 mL batch cultivation using shaking flasks growth resembled cultivation with PCA (data not shown). The production of siderophores has repeatedly been associated with *C. glutamicum*,^47,48^ and studies have shown the necessity of PCA or a comparable iron chelator in batch cultivation to reduce lag phases and accelerate growth in the first place.^36,37^ In batch culture the natural production of siderophores appears to be sufficient for growth initiation, while medium removal in continuous cultivation, such as within our microfluidic system, prevents it.

**Fig. 5:**
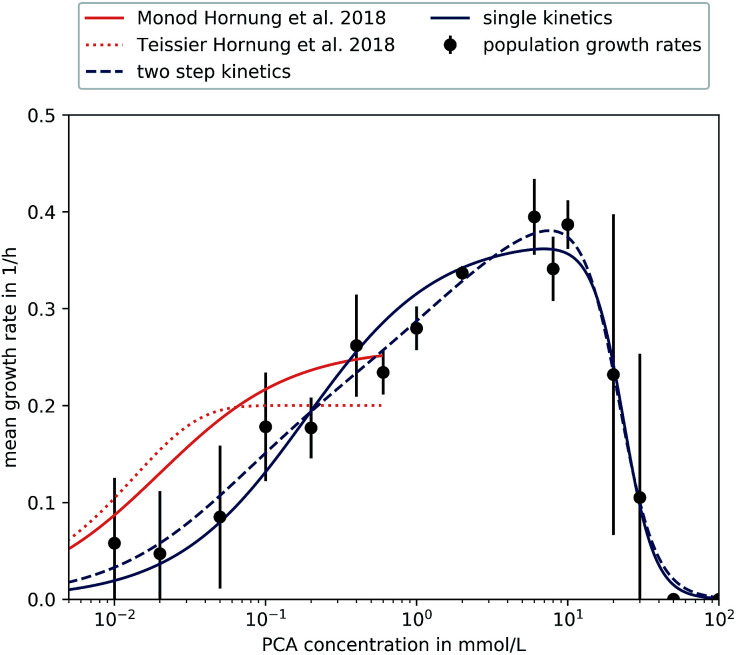
Different kinetics fitted to experimental results. Results found in microfluidic experiments by Hornung *et al.* have also been included.^16^ Error bars depict the standard deviation of population growth rates of biological replicates where more than two were evaluated (PCA concentrations over 0.05 mmol L^−1^).

Fig. 5 shows the mean growth rates for different PCA concentrations on half-logarithmic scale. The means are the average of the biological replicates for each concentration, where the population growth rate for a single experiment was determined from the distribution of single-cell generation times as described in Section 2.5.

The supply of PCA as sole carbon source and the subsequent observation of growth during microfluidic single-cell cultivation verified the reported findings that *C. glutamicum* is indeed capable of actively metabolizing PCA for its carbon demand. So far, PCA has been added to the nutrient medium as an iron chelator with a concentration of 0.195 mmol L^−1^. We could confirm earlier findings that 0.02 mmol L^−1^ PCA as sole carbon source leads to *μ* around 0.2 h^−1^ already.^37^ The maximum growth rate was observed at 6 mmol L^−1^ with a value of 0.4 ± 0.04 h^−1^ showing that the potential influence of PCA on growth is significant.

For PCA concentrations from 0.01 mmol L^−1^ to 0.05 mmol L^−1^, the cells showed very heterogeneous and slow growth, relatively independent of the applied concentration. As mentioned previously, heterogeneity was found along the length of the growth channels, expected to be caused by concentration gradients. Furthermore, slow growth and limited experiment time of 40 h resulted in an overall lower sample size compared to higher concentrations. Furthermore, for some experiments we did not find any division events at all, resulting in growth rates of 0.

A growth rate increase approximately proportional to the logarithm of the applied PCA concentration of 0.1 mmol L^−1^ to 0.4 mmol L^−1^ could be observed. Compared to 0.05 mmol L^−1^, nutrient supply seemed to exceed requirements for maintenance and more energy was put into proliferation and growth. This can be compared to results from earlier experiments by Hornung *et al.*^16^ They have investigated growth rates based on particle image velocity (PIV) and developed a simple analytic model to describe steady-state growth of cells in monolayer growth chambers. With the data from this study, we tried to corroborate and extend the existing growth model to higher concentration regimes.

The growth rates increased roughly as expected until 6 mmol L^−1^, where a maximum growth rate of (0.40 ± 0.04) h^−1^ was observed. Afterwards, growth rates in 20 mmol L^−1^, 30 mmol L^−1^, 50 mmol L^−1^ and 100 mmol L^−1^ PCA decreased in comparison to the observed *μ*_max_ at 6 mmol L^−1^, pointing towards an inhibitory effect of high PCA concentrations. For 30 mmol L^−1^ the results were very heterogeneous: for two experiments we observed comparatively fast growth around 0.3 h^−1^, while three other experiments with the same conditions did not yield any growth at all. Concentrations above 30 mmol L^−1^ PCA indicated bacteriostatic conditions due to toxicity of the nutrient itself or osmotic pressure. Haußmann and Poetsch elucidated the proteome response of *C. glutamicum* in modified MMES medium (sulfur-free minimal medium) with a PCA content of 100 mmol L^−1^ as sole carbon source.^49^ They found that the metabolization of such aromatic compounds was challenging due to their physico-chemical properties and toxicity in general. In more detail, reduction equivalents like ATP were rare, demanding the TCA cycle and oxidative phosphorylation to be activated for energy generation, marking PCA as a gluconeogenic carbon source. Additionally, growth on PCA seemed to reduce amino acid biosynthesis leading to carbon-starvation responses, an up-regulation of respiratory chain proteins, and significant alterations in cell wall biosynthesis.^49^

For a more comprehensive analysis, two different models have been introduced in Section 2.5, which were fitted to the experimental data to provide a mathematical description of the observed results. The experiments at very low concentrations, apart from experiments at 0 mmol L^−1^, were taken into account although we observed spatial heterogeneity. Both equations are based on a Monod-equation and assume PCA as sole growth limiting factor. To account for the observed growth stop at high concentrations the equations contain an inhibition term. Eqn (4) is a Monod-kinetic multiplied with the inhibition term, which can be interpreted as one limiting enzyme and an inhibitory effect of the substrate. Eqn (5) is based on the idea of two enzymes or pathways working in parallel, so that two Monod-type kinetics are added and then multiplied with the inhibition term. This is based on the understanding of PCA metabolism as reported so far: transport of PCA into the cell can take place passively, mediated by porins, or actively *via* transporters like PcaK, whereupon the PCA is degraded *via* the β-ketoadipate pathway.^49^ Transport efficiency of the PcaK transporter was so far only investigated in model systems of *E. coli* where a saturation appeared at 5 nmol mg^−1^ PcaK protein.^50^ If one assumes that the intracellular supply of PCA is ensured, another reason for stagnating growth may be the degrading pathway itself. Zhao *et al.* performed comprehensive studies on regulating mechanisms of the β-ketoadipate pathway, responsible for PCA degradation.^51^ In the course of their work, the authors found the positive regulatory protein PcaO, which responded to the presence of PCA by activating its degrading branch of the β-ketoadipate pathway. They further reported that PCA reduced the effect of PcaO in low concentrations (0.2 mmol L^−1^ and 0.5 mmol L^−1^) and supported its effect in higher concentrations (1 mmol L^−1^ and 2 mmol L^−1^).

However, both the single-step as well as the two-step kinetic are able to reproduce equally well the measured data, so that the parallel activity of two regulatory mechanisms could not be confirmed in this study. Furthermore, both differ from the results by Hornung *et al.*^16^ The reasons for this could be attributed to the very different experimental setup and the fact that our study did not account for spatial heterogeneity at low concentrations. Table 1 shows the estimated kinetic parameters for our study and the results by Hornung *et al.*^16^

**Table 1:** Parameters for different models fitted to the data of this study and from previous studies by Hornung *et al.*^16^ See Fig. 5 for the resulting growth kinetics

Model	*μ̂*_1_, h^−1^	*μ̂*_2_, h^−1^	*K*_1_, mmol L^−1^	*K*_2_, mmol L^−1^	*K*_I_, mmol L^−1^	*n*_I_
Single step	0.37	—	0.185	—	23.37	4.2
Two step	0.22	0.2	0.061	1.71	21.66	3.5
Monod^16^	0.26	—	0.020	—	—	
Teissier^16^	0.20	—	0.014	—	—	

### 3.3 Heterogeneity

**Fig. 6:**
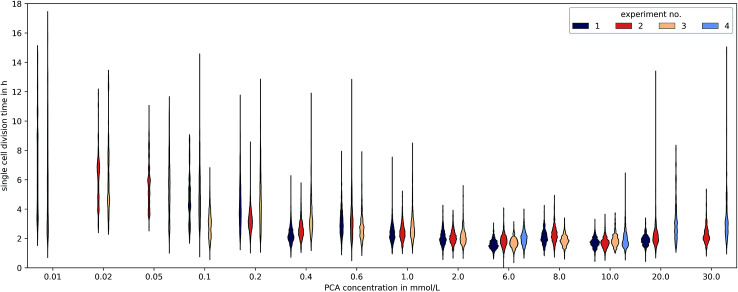
Distributions of single-cell generation times for different PCA concentrations and experiment replicates. Experiments without growth (*e.g.* at 100 mmol L^−1^) or less than 50 division events are not shown and only cell divisions starting 15 h after the beginning of the experiment are included.

The microfluidic approach allowed us to determine generation times on single-cell basis. Fig. 6 shows the division time distributions for individual experiments at different concentrations. PCA concentrations between 0.02 and 0.05 mmol L^−1^ displayed a wide spread of doubling times with a relatively high portion of cells that needed longer than 10 h for a complete division cycle. With increasing PCA concentrations (≥0.1 mmol L^−1^) the distribution sharpened and shifted towards shorter generation times. A minimum of heterogeneity was detected at the maximum growth rate in 6 mmol L^−1^ PCA.

**Fig. 7:**
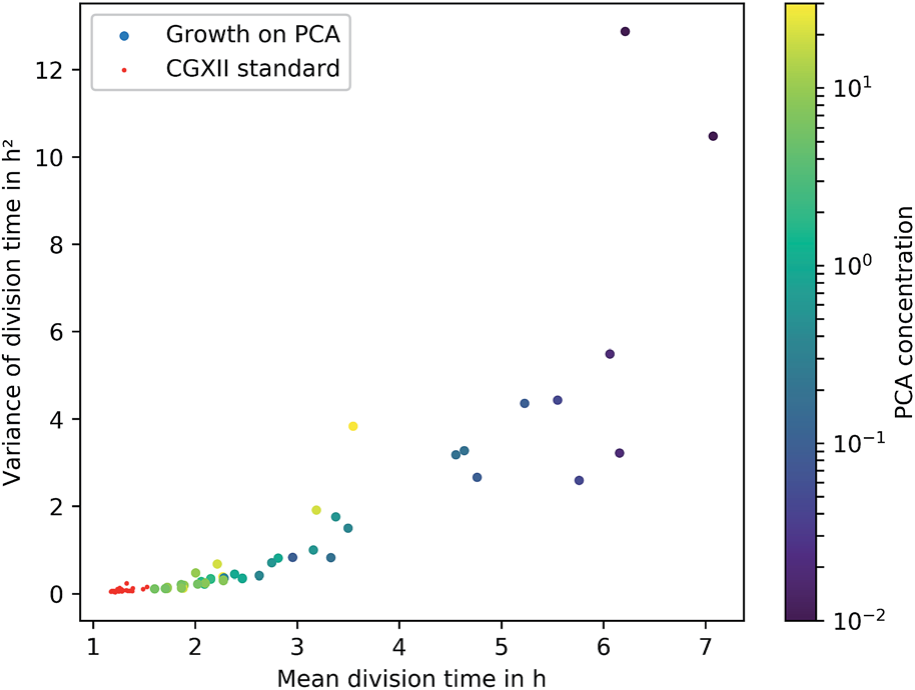
Variance over the mean division time for the different experiments. Experiments with less than 50 division events are not shown. The color of the dots shows the respective PCA concentration in mmol L^−1^. The red data points represent cultivations on the standard CGXII medium with 0.195 mmol L^−1^ PCA and glucose.

A common method to display heterogeneity in growth is to plot the variance against the respective mean generation time, Fig. 7 (see also Fig. 18, ESI† for the coefficient of variation). The results indicate that for *C. glutamicum* the population-wide generation time is positively correlated with the variance of the generation time distribution, implying an increase in heterogeneity and noise in low growth rates.

Various authors have reported similar observations for other organisms in varying environmental conditions and have proposed different explanations. Hashimoto *et al.* have reported a linear dependency for *E. coli* and found an *x*-intercept of this linear relation close to the minimum generation time within rich medium.^13^ De Martino *et al.* have developed a model employing entropy, which associates *μ*_max_ and the “inverse temperature” as variables for a fixed mean growth rate, showing the trade-off between dynamically favoured, fast phenotypes and entropically favoured, slow growing ones.^52^ Schreiber *et al.* have hypothesized an evolutionary advantage of heterogeneity in challenging environments by the simultaneous presence of various capabilities as phenotypes. They predicted an increase of phenotypic heterogeneity in microbial metabolism under nutrient-limited, dynamic habitats compared to nutrient-saturated, stable habitats.^28^ Even though these studies are based on other organisms, the observation of heterogeneous generation time distributions under low nutrient supply, comparable to *C. glutamicum*, hint towards similar underlying mechanisms. Furthermore, the present data reflect a re-occurrence of the phenomenon in PCA concentrations above 10 mmol L^−1^. The causing circumstances might be the challenging conditions due to osmotic pressure or toxicity, but this requires further work to be understood in greater detail.

## 4 Conclusions

In this study the growth effect of PCA in CGXII has been quantified for the full concentration range for the first time. A combination of two different microfluidic approaches, one developed previously by Hornung *et al.*^16^ and the one employed within this study, has allowed us to investigate the growth response of *C. glutamicum* for different concentrations of PCA in unprecedented detail and precision. Furthermore we covered a very broad range of concentrations within a single device, showing the superior capability of mother-machines for this type of investigation. The single-cell data also revealed a positive correlation between mean division time and the variance of the single-cell division time distribution for *C. glutamicum*, which has previously been shown for other organisms.

The presented data is able to resolve a growth kinetic with a saturation-like behaviour and shows how the growth dependence on nutrient availability can be described well by classic Monod-type kinetics although the regulatory mechanism for PCA metabolism is quite complex. However, the single-cell resolution reveals an increasing cell-to-cell heterogeneity, as the cells “struggle for life” under inhibiting or very low concentrations.

Studies within microfluidic devices become more common, and the used media are usually identical to those employed in macroscopic cultivations. The example of the defined CGXII medium shows how differences in the cultivation method can magnify effects by medium components not relevant at the macro-scale. This is caused by the change from batch-like conditions with higher cell densities within shake-flasks or similar devices to the microfluidic environment with conditions equivalent to a chemostat operating at very low cell densities. When the CGXII medium was used in the microfluidic environment it was found that PCA raised the growth of *C. glutamicum* by *ca.* 0.2 h^−1^, which is a half of the maximally observed rate of 0.4 h^−1^ for this medium in shake flasks or micro-well plate cultivations.^37^ In macroscopic experiments this growth supporting effect has not been detected. For a correct interpretation of results it will be important to ensure all medium components do not elicit similarly strong biological responses as PCA does in CGXII when a medium is transferred from macroscopic experiments.

## Conflicts of interest

There are no conflicts to declare.

## Supplementary Material

RA-009-C9RA02454A-s001
