# Supplementary material for: Microbial single-cell growth response at defined carbon limiting conditions
Source: RSC Adv. 2019 May 7;9(25):14040–50. doi: 10.1039/c9ra02454a (PMC9064036; doi:10.1039/c9ra02454a)
Supplement: RA-009-C9RA02454A-s001 [file RA-009-C9RA02454A-s001.pdf]

This journal is © The Royal Society of Chemistry 2019

Supplementary Information

Microbial Single-Cell Growth Response at Defined Carbon Limiting Conditions

Lindemann *et al*

A Chipdesign

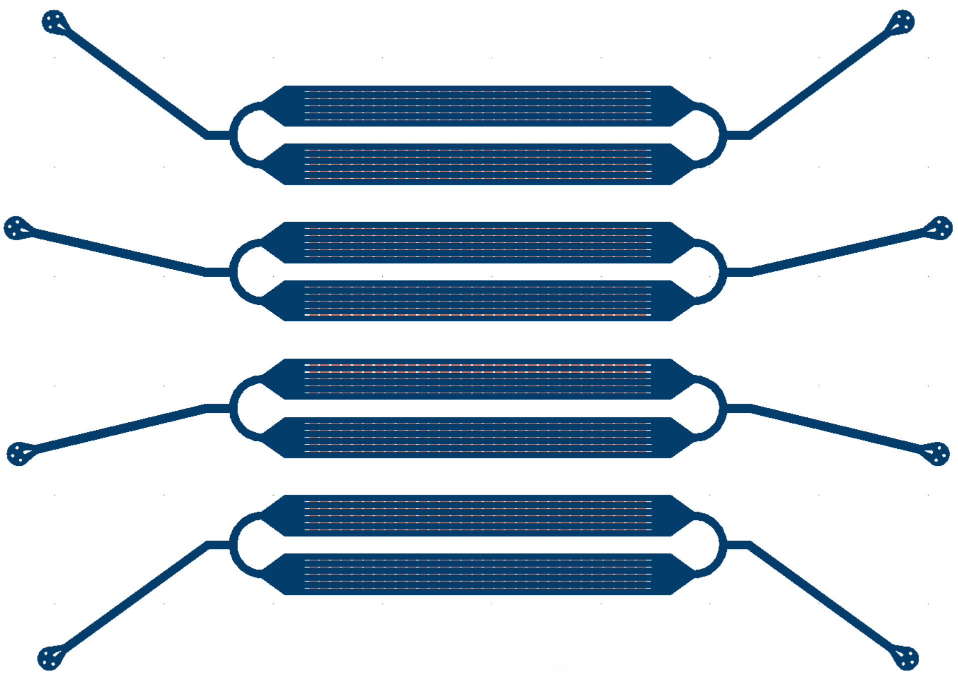

(a) Overview of a single chip with 4 different channel arrays.

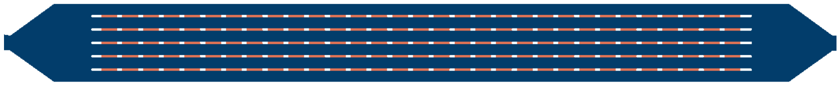

(b) Detail of the supply channels, each inlet splits into supply channels for 2 times 5 rows of 31 growth channel arrays each.

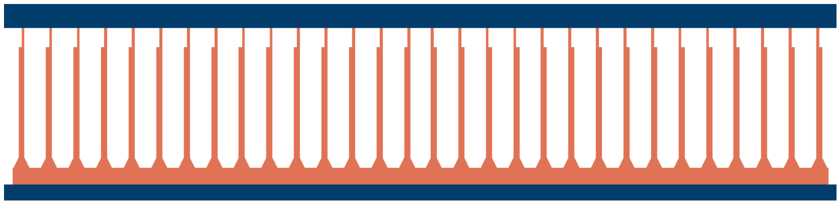

(c) Array of 30 growth channels array with one blocked end.

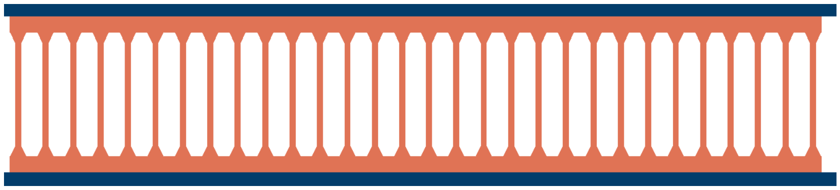

(d) Array of 30 growth channels with two open ends.

Fig. 8: Design of the microfluidic chips. The deep layer (dark blue) is ca. $10.8\,\mu\text{m}$ high, the shallow growth channel layer (orange) is ca. $0.8\,\mu\text{m}$ high.

B Estimating Concentrations Along Growth Channels

A simple calculation allows to estimate the concentration in the center of the channel, when it is filled with growing cells. The used growth channels are open at both ends, and for the purpose of the calculation the differences between the two types are ignored (see figure 8). Diffusion along a simple channel can be modelled with the following differential equation:

$$D\frac{d^2c}{dy^2} = \hat{U} \tag{9}$$

where D is the diffusion coefficient of the solute in the chamber in $\mathrm{m^2/s}$, c the nutrient concentration in $\mathrm{mol/L}$, y the spatial coordinate along the growth channel in m, and $\hat{U}$ the nutrient uptake per volume and time within the chamber in $\mathrm{mol/(m^3\,s)}$. Here we assume that the uptake is independent of concentration, and the cells in the channel do not block diffusion. If we integrate the equation two times we get:

$$c(y) = \frac{\hat{U}}{2D}y^2 + k_1 y + k_2 \tag{10}$$

where k_1 and k_2 are the integration constants. We know that the concentration at the connection between chamber and channel ($y = 0$) is equal to the concentration in the channel c_0. Therefore $k_2 = c_0$. We also fix the derivative of the concentration at $y = l/2$ to be zero because there is no further diffusive flow across the symmetry boundary in the middle of the channel with length l.

$$0 = \frac{dc}{dy} \tag{11}$$

$$0 = \frac{\hat{U}}{D}\frac{l}{2} + k_1 \tag{12}$$

$$k_1 = -\frac{\hat{U}l}{2D} \tag{13}$$

The equation for concentration in y-direction is then:

$$c(y) = \frac{\hat{U}}{2D}(y^2 - ly) + c_0 \tag{14}$$

Finally, to calculate the inlet concentration c_0, for which the concentration at $y = l/2$ is equal to $c_0/2$, we can use the following equation:

$$\frac{c_0}{2} = \frac{\hat{U}}{2D}(\frac{l^2}{4} - \frac{l^2}{2}) + c_0 \tag{15}$$

$$-\frac{c_0}{2} = -\frac{\hat{U}}{2D}\frac{l^2}{4} \tag{16}$$

$$c_0 = \frac{\hat{U}l^2}{4D} \tag{17}$$

C Additional Results

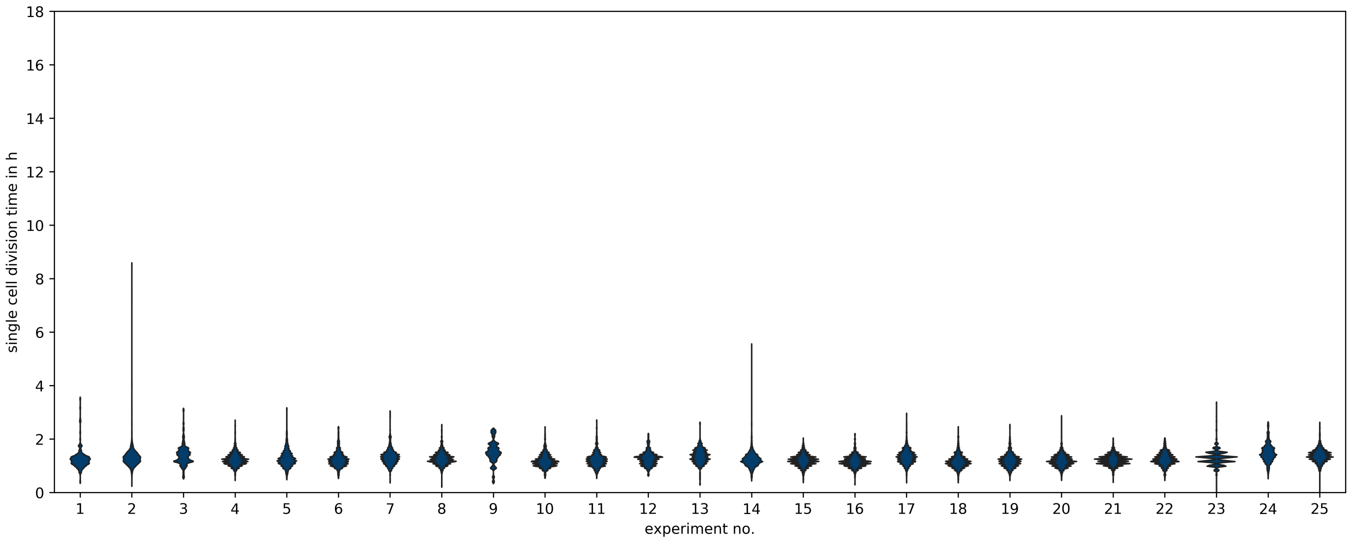

Fig. 9: Distributions of single-cell generation times for different experiment replicates of the standard CGXII with $4\,\%$ glucose and $0.2\,\mathrm{mmol/L}$ PCA. Only cell divisions starting $15\,\mathrm{h}$ after the beginning of the experiment are included. The experiment number 23 has been conducted with longer time between the positions than the other experiments.

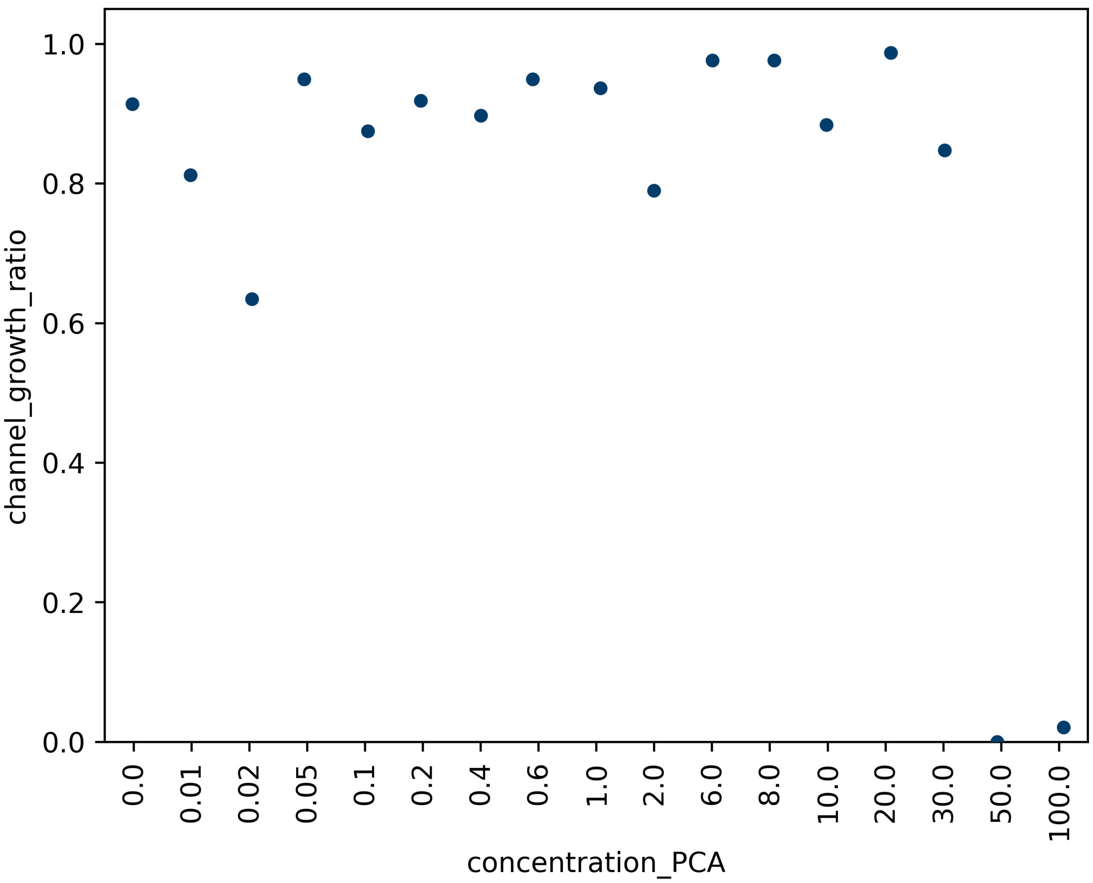

Fig. 10: Fraction of channels seeded with growing channels of all seeded channels for different PCA concentrations, aggregated from the single position data (see figure 11). The two colors distinguish both types of growth channels.

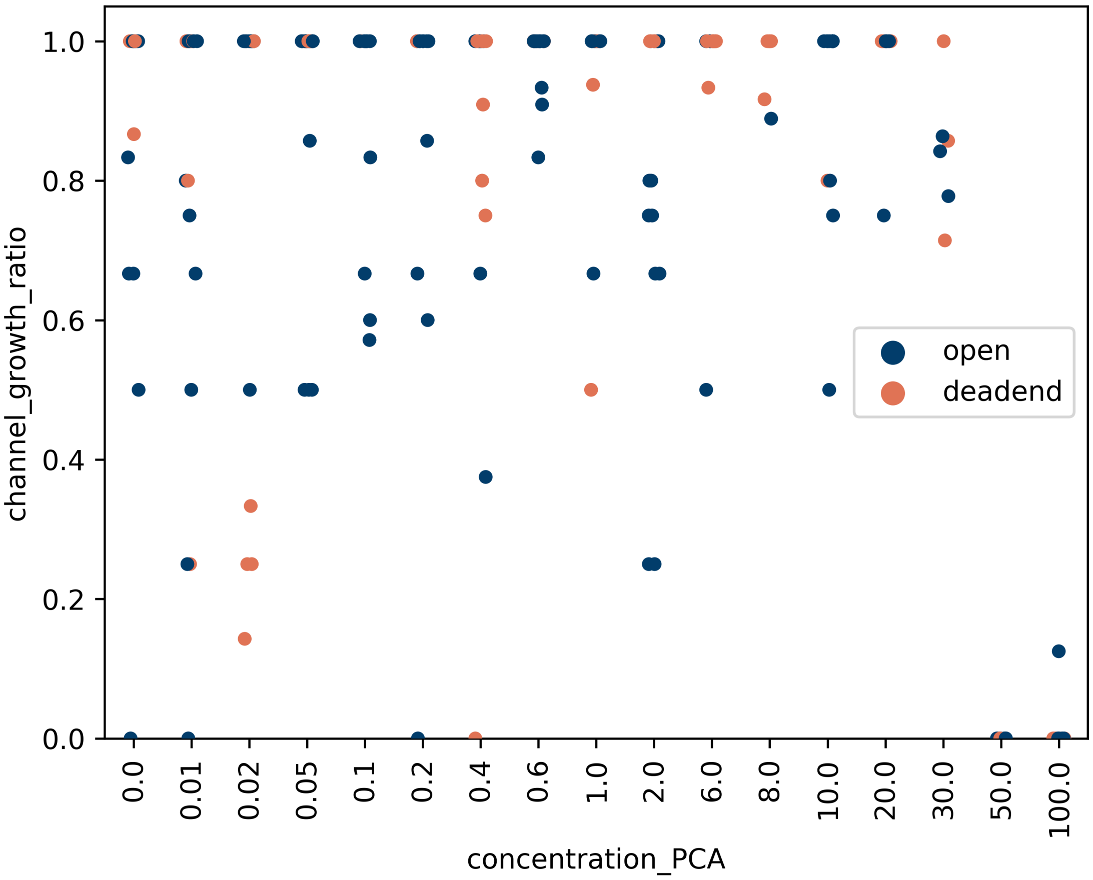

Fig. 11: Fraction of channels seeded with growing channels of all seeded channels for different PCA concentrations for the different observed positions. The two colors distinguish both types of growth channels.

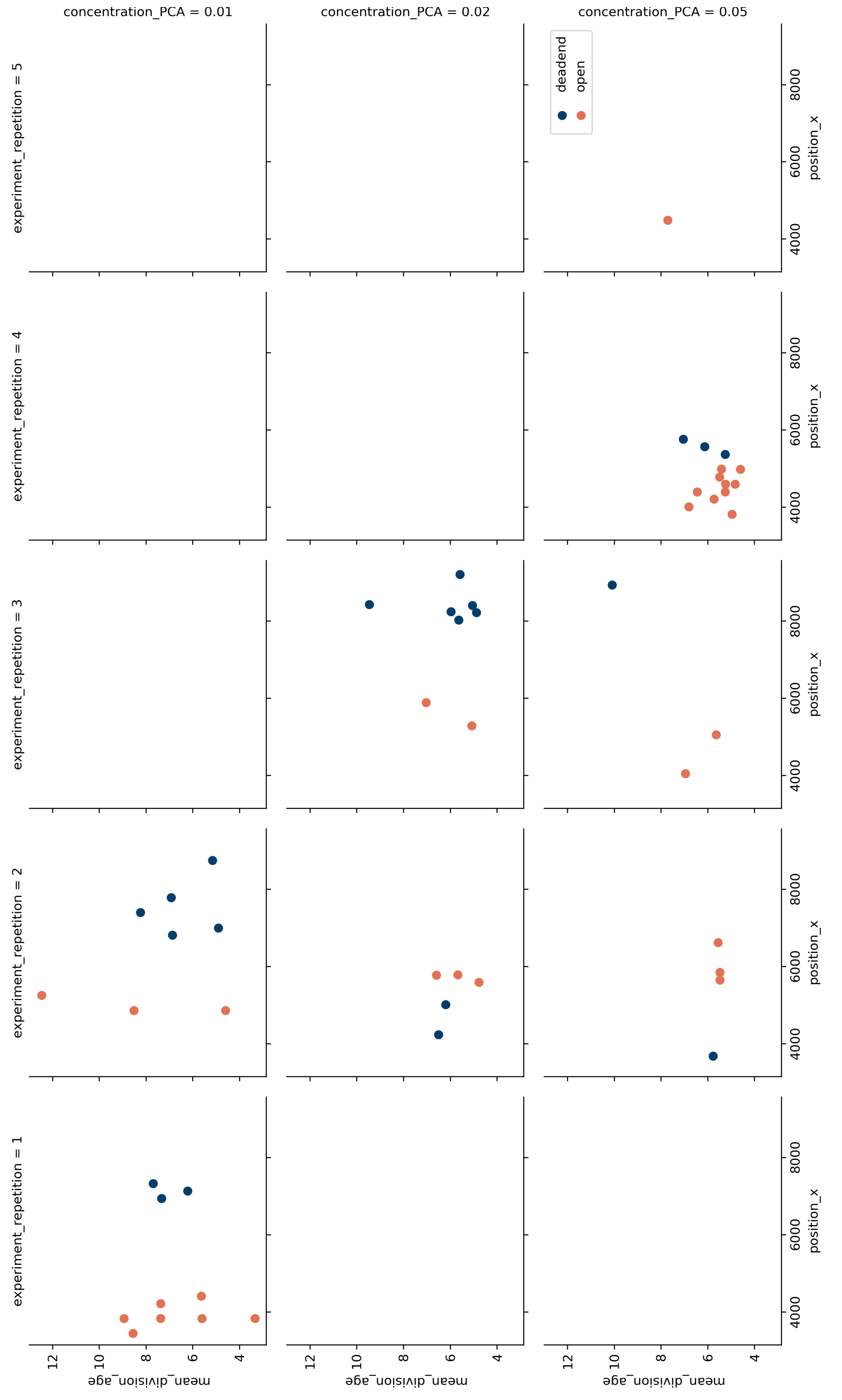

Fig. 12: Mean division time of different positions over their x position on the chip along the supply channels. The two colors distinguish both types of growth channels.

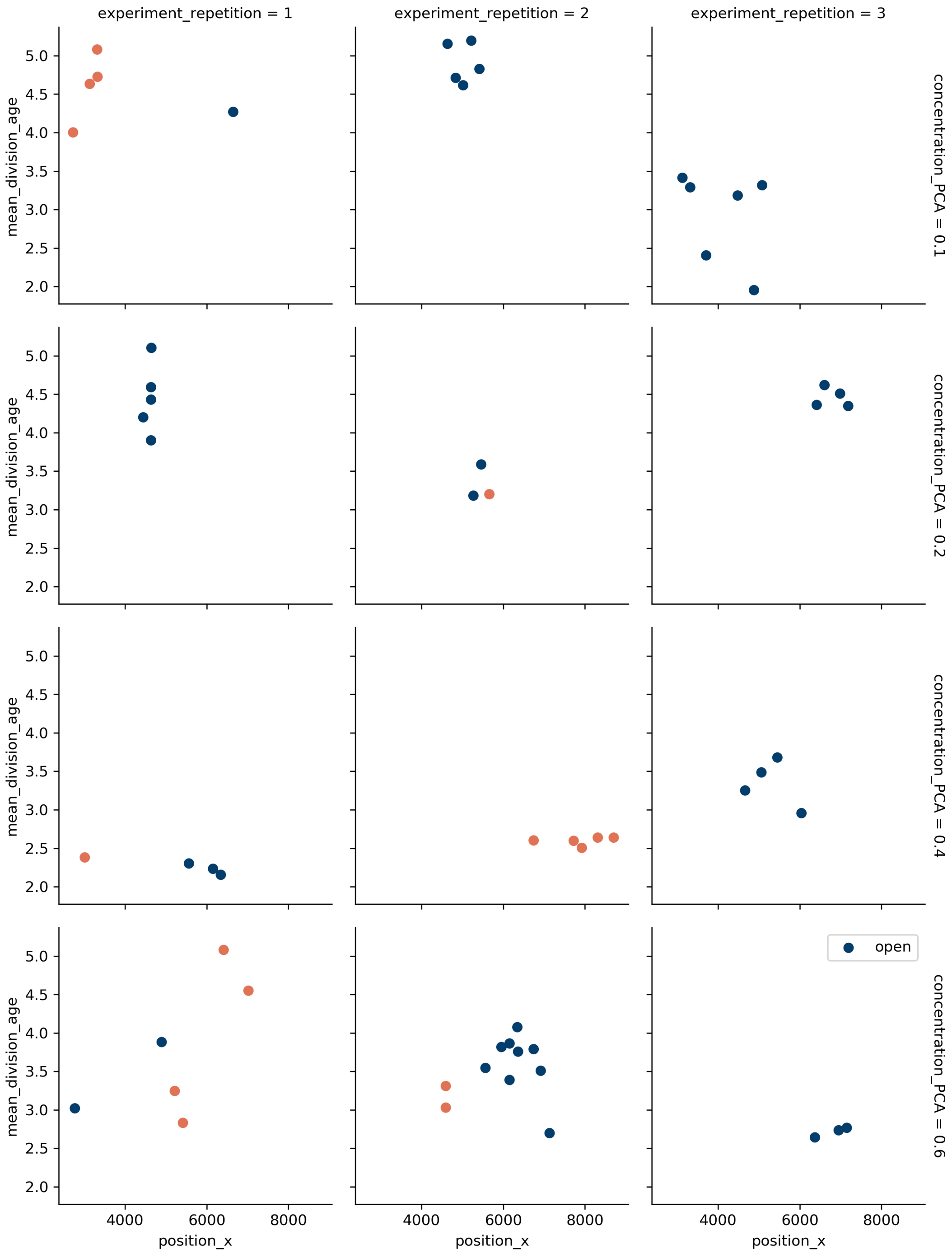

Fig. 13: Mean division time of different positions over their x position on the chip along the supply channels. The two colors distinguish both types of growth channels.

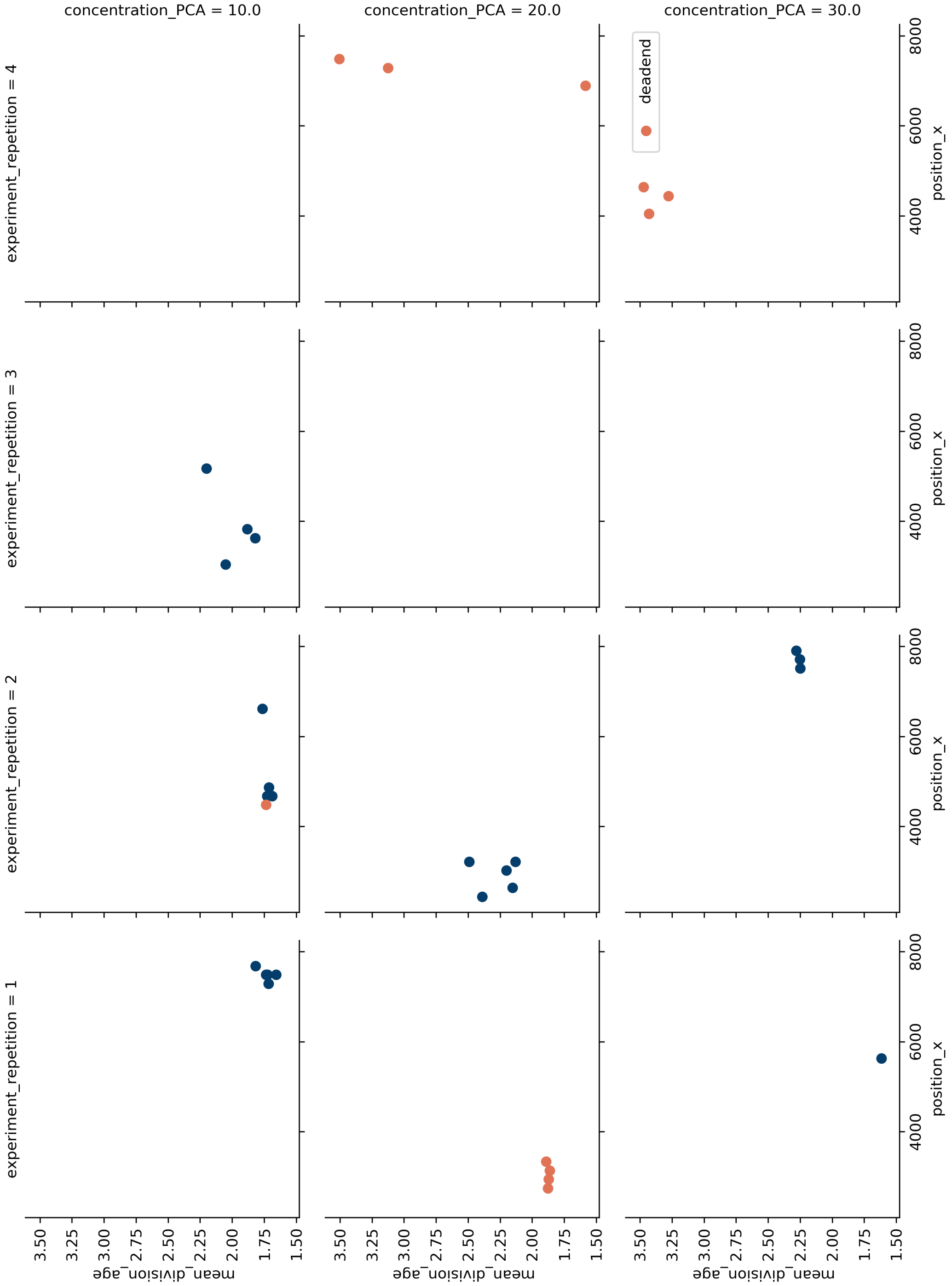

Fig. 14: Mean division time of different positions over their x position on the chip along the supply channels. The two colors distinguish both types of growth channels.

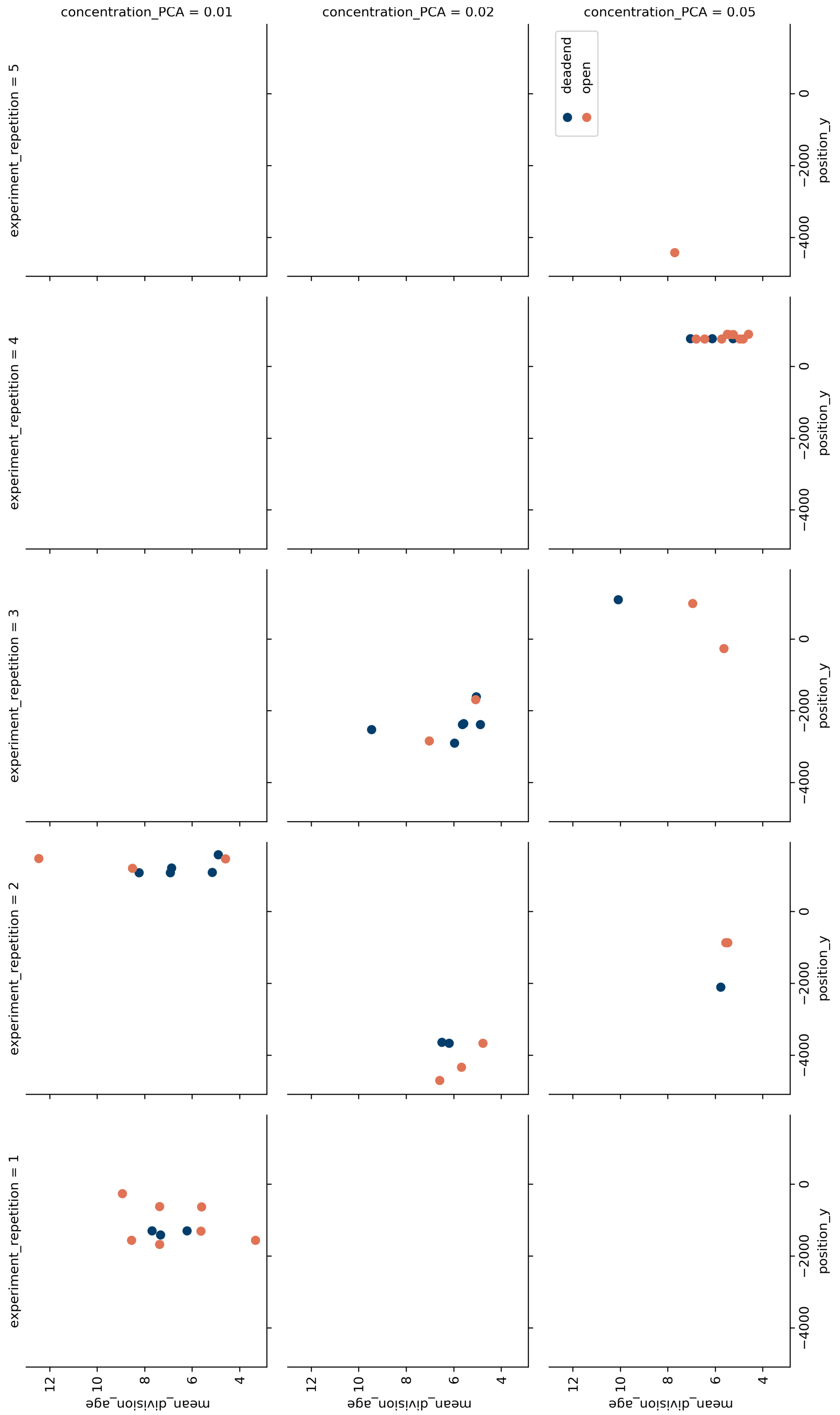

Fig. 15: Mean division time of different positions over their y position on the chip in the direction orthogonal to the supply channels. The two colors distinguish both types of growth channels.

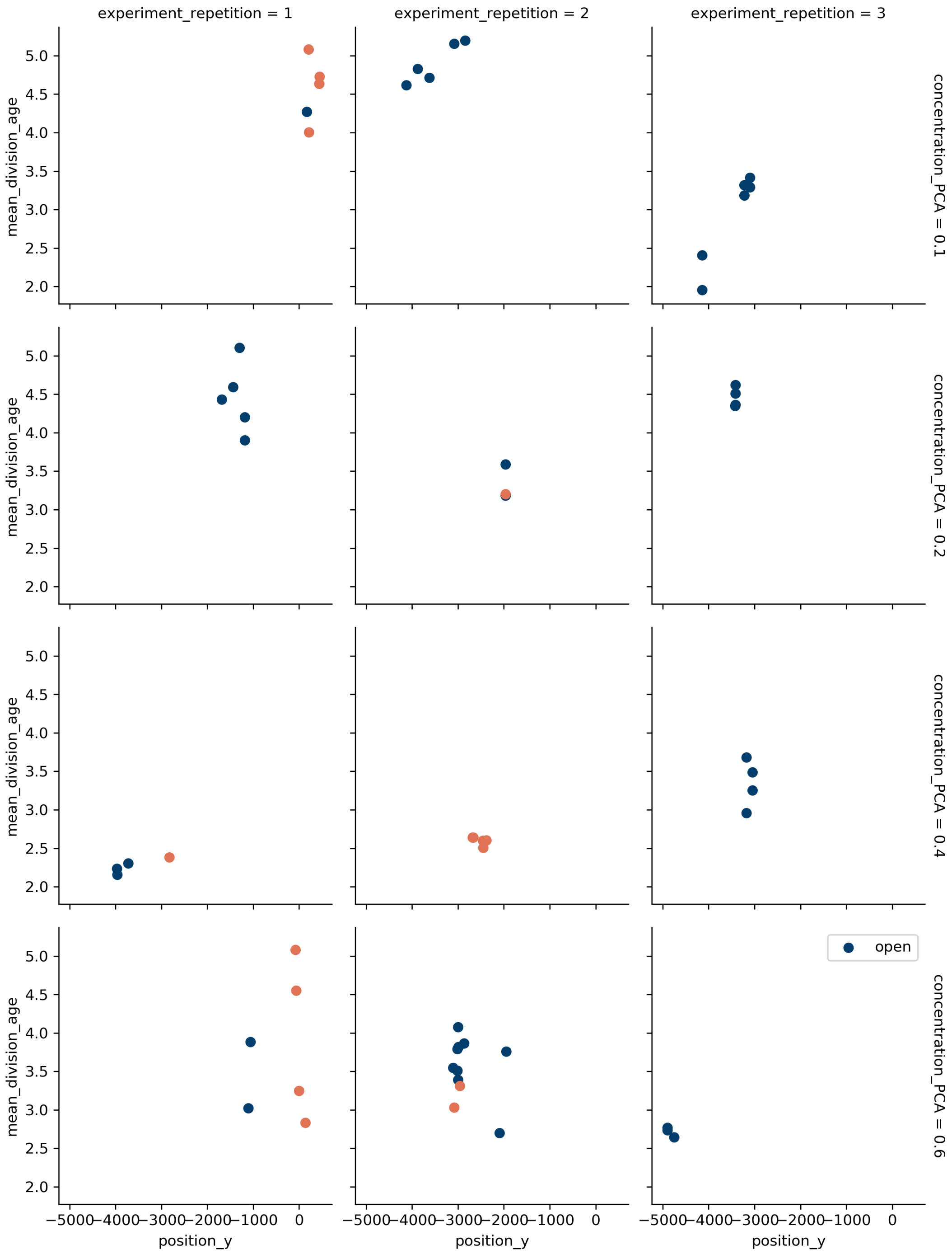

Fig. 16: Mean division time of different positions over their y position on the chip in the direction orthogonal to the supply channels. The two colors distinguish both types of growth channels.

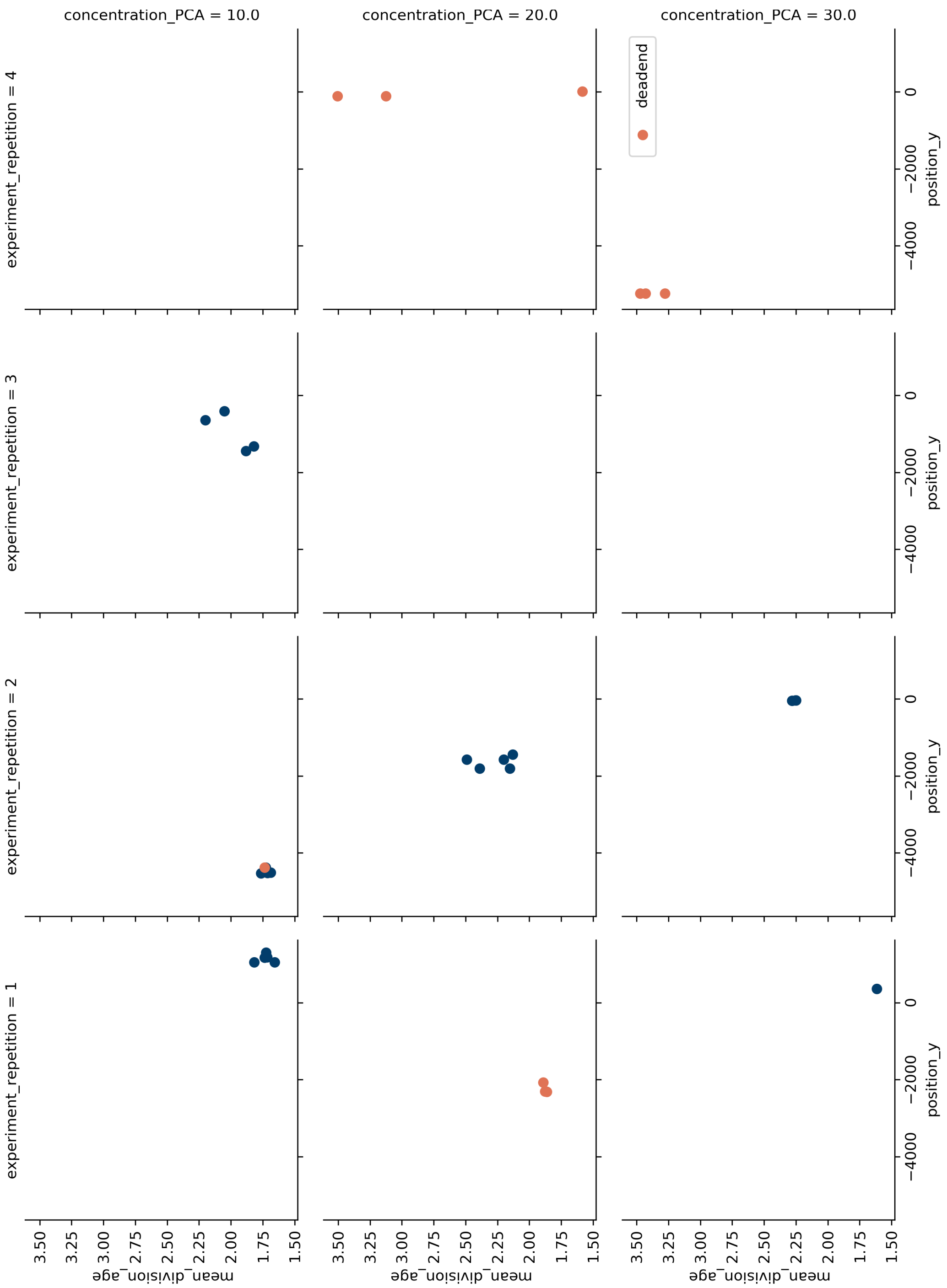

Fig. 17: Mean division time of different positions over their y position on the chip in the direction orthogonal to the supply channels. The two colors distinguish both types of growth channels.

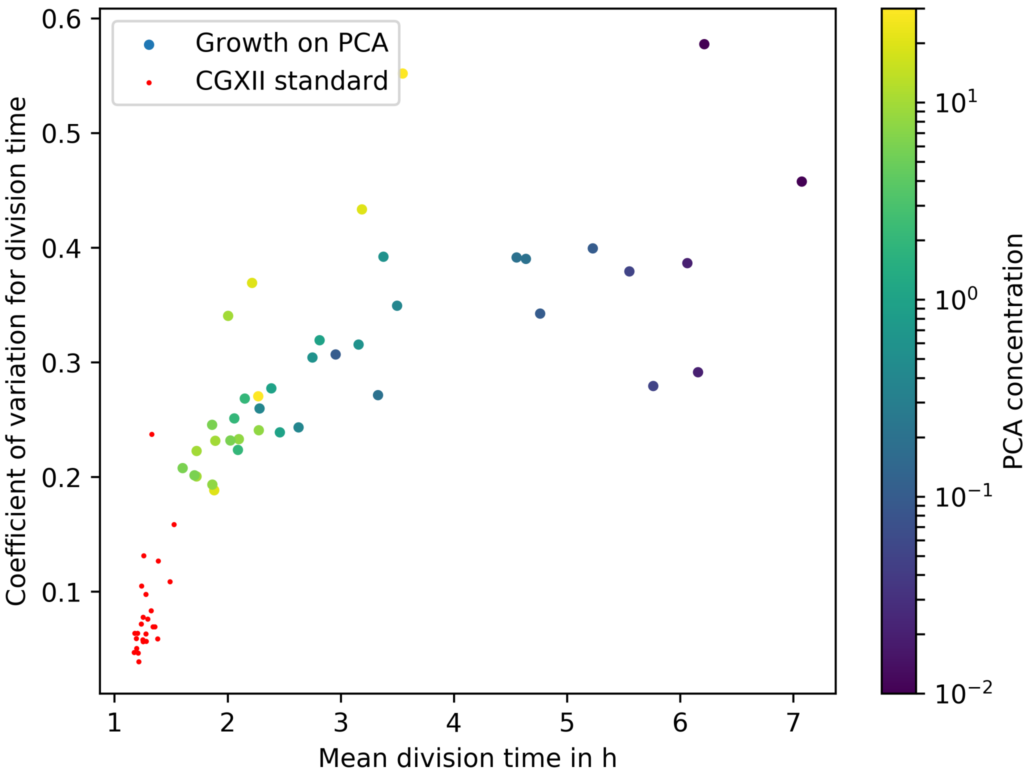

Fig. 18: Coefficient of variation over the mean division time for the different experiments. Experiments with less than 50 division events are not shown. The color of the dots shows the respective PCA concentration in $\mathrm{mmol/L}$. The red data points represent cultivations on the standard CGXII medium with $0.195\,\mathrm{mmol/L}$ PCA and glucose.
